# Spatiotemporal variations in migratory bird diversity and abundance along the coast of Gochang getbol

**DOI:** 10.1371/journal.pone.0300353

**Published:** 2024-05-31

**Authors:** Myung-Bok Lee, Ju-Hyun Lee, Gi-Chang Bing, Won-Suk Choi, Jung-Moon Ha, Jae-Ung Jang, Se-Yeong Kim, Jong-Ju Son, Ah-Jin Chang, Ji-Young Lee, Dae-Han Cho, Ha-Cheol Sung

**Affiliations:** 1 Department of Biological Sciences, Chonnam National University, Gwangju, Republic of Korea; 2 HNI, Sejong, Republic of Korea; 3 World Heritage Promotion Team of Korean Tidal Flats, Muan, Republic of Korea; 4 School of Biological Science, Seoul National University, Seoul, Republic of Korea; 5 School of Biological Sciences and Biotechnology, Chonnam National University, Gwangju, Republic of Korea; Liverpool John Moores University, UNITED KINGDOM

## Abstract

Tidal flats provide critical habitat for migratory waterbird species; however, populations of migratory waterbirds have significantly declined due to tidal flat loss and degradation caused by human activities, particularly in Asia. Gochang getbol is one of tidal flats located on the southwest coast of South Korea and a center of clam production. Using bird monitoring data collected at five zones (zone1 to zone5) established across Gochang getbol and near coastal area, we examined distribution patterns of migratory bird diversity and conservation-related species along the coast of Gochang getbol. The intensity of human activity ‒ mudflat culture (mostly bivalve) and aquaculture was relatively high at zone2 and zone3, occupying > 30% of 2km circular area surrounding most sample points of these zones. Zone1 and particularly zone4 contained more natural/semi-natural habitats (less disturbed mudflats and wetlands) and zone5 had smallest mudflat than others. Shannon diversity, species richness, and abundance of migratory birds differed between zones (Anova test, *P* ≤ 0.02) except Shannon diversity in winter. In fall, all values were higher at zone4 than zone3 and zone5. In winter, zone1 showed greatest species richness and higher abundance than zone2, zone3, and zone5. In spring, while most differences were found between zone4 and zone5, abundance at zone4 was somewhat higher than zone2. The results from the fourth corner analysis indicated that abundance of species foraging at mudflat level was positively associated with zone1 (winter) but negatively with zone3 (fall). Sandpipers were positively associated with zone4. Abundance distribution maps of conservation-related species, created by inverse distance-weighted interpolation modeling, also showed high abundance of most conservation-related species at zone4 and 1. The findings of our study suggest the importance of natural/semi-natural habitat, and the possible link between human activity and distribution patterns of migratory birds in Gochang getbol. While we need further investigation on direct response of migratory birds to human activity, areas with low human activity with more natural/semi-natural habitat, e.g., zone4 and zone1 may be crucial for the conservation of migratory birds.

## Introduction

Tidal flats (mudflats), occupying over 127,921km^2^ of the Earth’s surface [1], have a dynamic structure as they undergo regular cycle of exposure and inundation caused by tides with seasonal changes [2]. Although tidal flats often appear vegetationally deficient, they provide habitats for waterbirds and numerous benthic animals as well as important ecosystem services for millions of people globally [3, 4]. Tidal flats are of particular critical foraging sites of migratory waterbirds: for example, many tidal flats support over 100,000 shorebirds yearly [5].

However, in Asia where 44% of global tidal flats are located [1], diversity and population of migratory waterbirds have substantially declined due to tidal flat loss and change by human activities, i.e., coastal development and reclamation, aquaculture, mudflat culture (the culture of commercial mollusks and other species on mudflats), fishing, and so on [6–9]. Approximately 16.02% of tidal flats were lost during 1984 and 2016 with highest loss in Asia [1]. Tidal flat areas of Asia have been under significant human pressure due to high coastal human population [10] as well as intense coastal and offshore fisheries and aquaculture productions [11].

Migratory bird species are often grouped into 8 major flyways to facilitate international conservation effort according to species’ geographical occurrence and migration routes between breeding and non-breeding areas [12]. The East Asian-Australasian Flyway (EAAF) includes over 20 countries, spanning from Australia and New Zealand through East and Southeast Asia to Artic regions in Russia and Alaska. Of 8 flyways, EAAF has experienced the most significant decline in populations of migratory waterbirds, specifically at intertidal flats in the Yellow Sea lying between mainland China and the Korean peninsula due to habitat loss and environmental degradation [13–17]. Despite increasing conservation and restoration efforts during the past 10 years, tidal flats in the Yellow Sea have continued to degrade [18]. Fourteen populations of globally threatened or near-threatened migratory birds are still declining in the Yellow Sea [18].

The tidal flats along the west coastline of South Korea (the Republic of Korea) are well-known wintering and staging sites of migratory birds in EAAF [13]. However, over 65% of tidal flats have been lost during the last half of the 20th century [14] with the greatest loss rate (-1.58% per year) in EAFF between 1980s and 2000s [19]. With increasing awareness on the ecological and economic values of tidal flats, the South Korean government has enacted Wetlands Conservation Act in 1999 and expanded marine protected areas to preserve the tidal flats [20, 21]. About a half of the remaining tidal flats in South Korea are situated in Jella Province. One of tidal flats in the province, Gochang getbol is a center of clam production in South Korea, supplying 40–50% of domestic Manila clam (*Ruditapes philippinarum*) production [22, 23]. Gochang getbol has unique geological structure, called Chenier and complex sediment types with rich benthic macroinvertebrates [24]. Gochang getbol has been recently designated as the World Natural Heritage site, acknowledging the important value of the mudflat ecosystem of the getbol. However, there is little scientific knowledge on the distribution pattern of migrant bird diversity and populations within Gochang getbol and possible impact of human activity (bivalve culture and aquaculture pond) that may lead to mudflat loss and decrease in habitat quality of mudflat. Understanding spatial and temporal variations in migrant birds in the getbol area and relevant environmental characteristics could be critical not only to maintain the status of the World Natural Heritage site of the getbol but also to develop effective conservation plan for migrant birds in EAAF [25].

Here, we investigated the distribution patterns of migrant birds along the coastline of Gochang getbol and near area, which show variations in environmental characteristics, especially the amount of natural/semi-natural habitat (e.g., mudflat and wetland) and the intensity of human activity (resource use by human including bivalve culture and aquaculture pond for shrimp farming). Our study was primarily interested in understanding 1) spatiotemporal pattern of species diversity and abundance of migratory birds in Gochang getbol, and 2) abundance distribution pattern of conservation-related species (i.e., globally and/or nationally protected species). By understanding these patterns, we also aimed to identify a key site for the conservation of migratory birds in the getbol and describe potential environmental characteristics associated with the patterns. It is expected that abundance of conservation-related species as well as migrant bird diversity and abundance could be higher at less disturbed sites (low human activity, i.e., low bivalve culture and aquaculture) with more natural/semi-natural habitats (e.g., mudflats and wetlands) [17, 26–28; but see 29, 30]. Spatial distribution patterns of migratory birds are also likely related to species’ traits (e.g., body mass, foraging stratum, and family) because species’ traits can mediate species’ responses to disturbance and determine species occurrence in a habitat by interacting with environmental characteristics of the habitats [31, 32]. However, the patterns may be inconsistent between seasons, especially winter and spring/fall due to variations in species composition.

## Methods

### Ethics statement

All field data collected in our study were observational: counting birds by sight and sound (see “Bird data” below). We confirm that our study did not involve the handling of any animal, which did not require approval from an Institutional Animal Care and Use Committee or equivalent animal ethics committee.

### Study area and design

**Fig 1:**
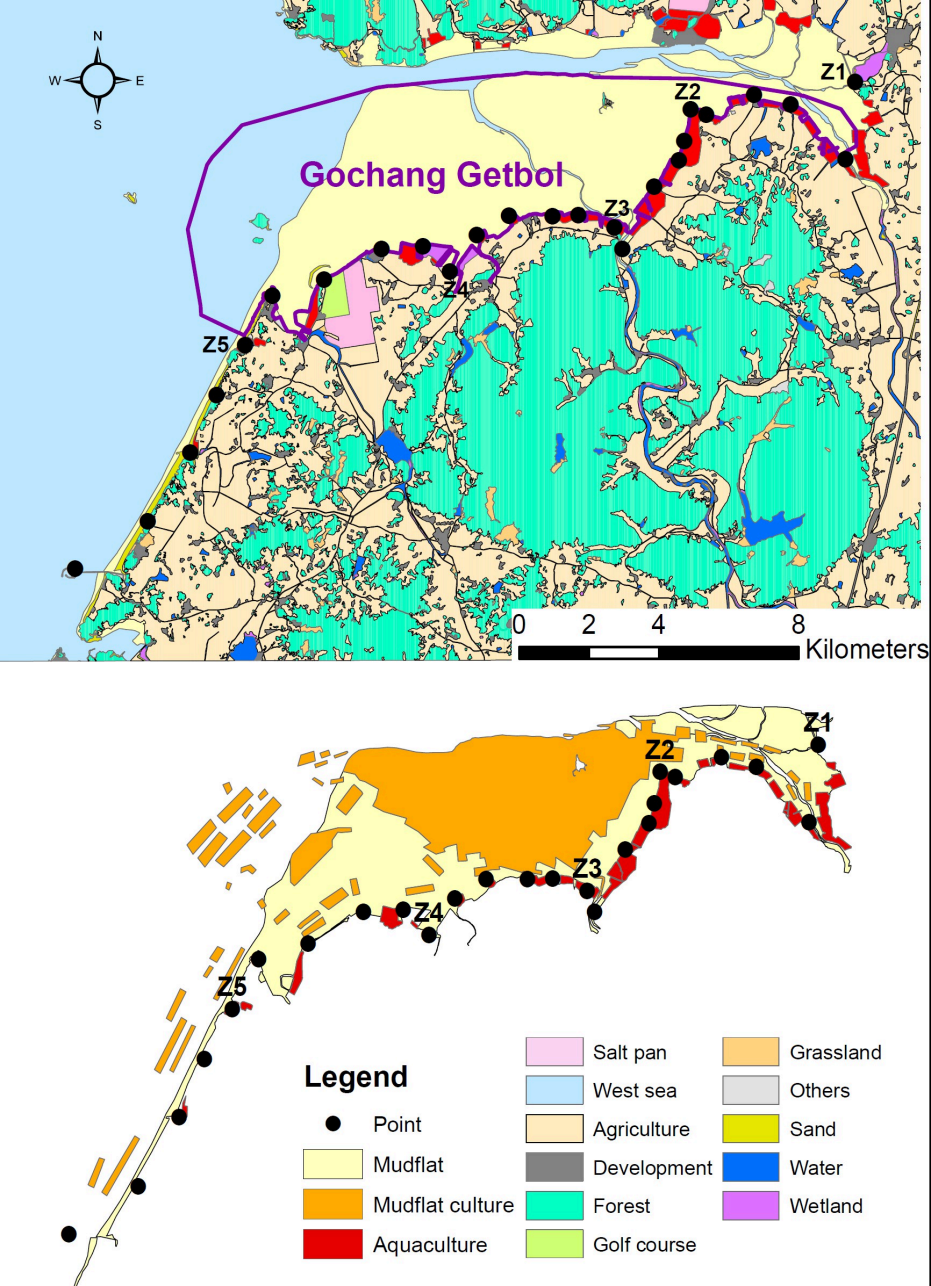
Location of sample points (top) and the intensity of human activity (mudflat culture‒mostly bivalve culture, and aquaculture; bottom) along the coast of Gochang getbol and near area. The first point of each zone was denoted by Z with zone number: for example, Z1 represents the first point of zone1.

Gochang getbol is an open-embayed mudflat situated in the southwestern coast of the Korean peninsula (35˚32´56.54˝N, 126˚32´59.75˝E), covering 5,531ha of the coastal area (Fig 1). It is one of four tidal flats designated World Natural Heritage sites in South Korea. Large portion of the getbol (4,550 ha) is also enlisted in the Ramsar Sites. About 0.96 km^2^ and 0.39 km^2^ areas of the getbol have been restored during 2010–2013 and 2017–2020, respectively. Port construction and particularly aquaculture pond (shrimp farming) noticeably increased along the coast of Gochang getbol during 2000’s (S1 Fig).

Dominant benthic macroinvertebrates other than Manila clam are black clam (*Cyclina sinensis*) and *Ruditapes philippinarum*, followed by Korean mud snail (*Bullacta exarata*) and intertidal clam (*Mactra veneriformis*) at the central area in the getbol [33]. Capitellid threadworm (*Heteromastus filiformis*) and *Scolelepis sp*. are also common [34]. While mudflat culture/harvest (largely bivalves; bivalve culture, hereafter) occurs across the getbol, it is concentrated on the central area (Fig 1). Climate of the region is “warm and temperate” with a mean annual temperature of 12.7°C (highest in August [25.2°C] and coldest in January [-0.0°C]) and annual precipitation of ~ 1222 mm [35].

We divided Gochang getbol and near coastal area into 5 zones, and established 5 points at each zone systematically by considering accessibility (Fig 1). Mean distance between points was 1.20km (± 0.57km standard deviation, ranging from 0.46km to 2.47km); however, the 5^th^ and 1^st^ points of some neighboring zones were close, < 0.5km far apart. The 5^th^ point of zone2 was located in an estuary. Sediment type varied across our study sites, showing a gradient of decreasing silt and clay with increasing sand from zone1 to zone5 [36, 37].

We initially determined the 5 zones not only to facilitate our surveys but also to account for different level of human activity (the size of area allowed for bivalve culture and aquaculture) by referring marine resource and property management map of Gomso Bay. The map was acquired from Gochang-gun and yet opened to public. To verify variations in the intensity of human activity between zones, we digitized the map without individual property types and boundaries, and incorporated it into land cover map generated by modifying the national land cover database (LandCoverMap; [38]). We then calculated percent covers of bivalve culture, aquaculture pond, mudflat (less disturbed mudflat, i.e., mudflat area at which little human activity occurs), and other landscape elements within a 2km circular buffer surrounding a sample point. The size of buffer was somewhat arbitrary but large enough to include all bird detections and characterize matrices of any points within a zone. The intensity of human activity was relatively similar between points within a zone, except the 5^th^ point of zone1, zone2, and zone3 (S1 Table) but differed between zones (Table 1). Of 5 zones, zone2 and zone3 showed the highest human activity with a mean percent cover of 36–38. Although bivalve culture was dominant at both zones, aquaculture ponds were common at zone2 (Table 1 and Fig 1). Large portion of zone1 and 4 was covered with less disturbed mudflats. Both zones, particularly zone4 had highest amount of other natural/semi-natural habitats such as old salt pan and wetland (restored mudflat). While zone5 showed low level of human activity, the mudflat of zone5 was very small and sand-dominant sediment [36, 37].

**Table 1:** Relative percentage (mean ± standard deviation) of main land covers that could be associated with migratory waterbird distribution in Gochang getbol. The values are calculated from the first four points of each zone (See S1 Table for the percentage of land covers at each point). Mudflat represents less disturbed mudflat which experiences little human activities.

	Human activity		Natural/semi-natural habitat		
Zone	Aquaculture	Bivalve culture	Mudflat	Salt pan	Wetland
zone1	6.37 ± 2.05	4.74 ± 3.25	38.35 ± 14.54	0 ± 0	1.10 ± 1.84
zone2	10.66 ± 1.89	27.10 ± 5.13	24.72 ± 2.74	0 ± 0	0 ± 0
zone3	5.25 ± 2.50	31.06 ± 9.77	18.29 ± 4.87	0 ± 0	0.37 ± 0.64
zone4	2.51 ± 0.56	4.31 ± 1.76	42.77 ± 11.27	6.51 ± 7.22	2.23 ± 1.59
zone5	0.69 ± 0.56	4.62 ± 1.51	6.95 ± 4	0 ± 0	0.04 ± 0.04

Major part of Gochang getbol contains the area covering from the 3^rd^ point of zone1 to the 1^st^ point of zone5. We included the 1^st^ and the 2^nd^ point of zone1 because they are protected as the Ramsar Sites and within a buffer area of the getbol. All points of zone5 were also surveyed for comparisons and monitoring purpose due to the occurrence of globally endangered species, Chinese crested tern (*Thalasseus bernsteini*).

### Bird data

During mid-August 2022 and May 2023, bird surveys were performed by two teams of observers simultaneously to avoid double-counting following the survey protocol of Wetlands International [39] approximately every two weeks, resulting in a total of 20 surveys. At each point, observers scanned the tidal flat area < ~1km in distance from them, and identified and counted all birds seen using binoculars and a telescope. We alternated observers between surveys to minimize observer effect on bird detection. All surveys were conducted 2 hours before or after high tide (mostly before high tide). At sites with large mudflat areas like our study site, it is recommended “counting tidal areas on a rising tide” when the size of mudflat area is reduced and so birds are within identification range [39].

We grouped birds detected according to their migratory status: summer visitor, passenger, and winter visitor (S2 Table for species list). We excluded resident species for community analysis because only 7 of 84 species observed were resident birds, composing a small portion of bird community at the getbol. In general, summer visitors arrive in South Korea in March/April and leave during August–October after breeding. Passengers’ northward migration and southward migration peak in April–May and September–October, respectively. Winter visitors appear in October and increase till December/January. We considered both passengers and summer visitors for the analysis of fall and spring seasons and winter visitors for the analysis of winter season.

When conservation-related species (Csp; residents as well as migrants but mainly migrants) was found during each survey, we collected data on not only number but also GPS location of the Csp. We also recorded number and GPS location of Csp that were observed while travelling from point to point. In addition to 20 surveys, three more surveys (late-July 2022, late-May, and mid-June 2023) were conducted for Csp using the same method, resulting in a total of 23 surveys.

### Data analysis

#### Community level analysis

The bird data were pulled together because of low detection and to minimize spatial dependency in the data. Within each zone, we excluded the 5^th^ point and combined other points into two pairs in order: the 1^st^ and 2^nd^ points into the one pair and the 3^rd^ and 4^th^ points into the other pair. These pairs were used as sample sites for analysis.

We divided the survey periods into three seasons, fall (mid-August–October 2022), winter (mid-November 2022 –January 2023), and spring (mid-March–May 2023) based on the time of migration and variations in bird composition. To avoid potential bias associated with transitional patterns between seasons, we excluded 2 surveys conducted in late October and early November 2022, and 3 surveys in February and early March 2023. Each season had 5 surveys for analysis. We used 37 species for fall season, 38 species for winter season, and 31 species for spring season analyses (S2 Table). Two species diversity indices, i.e., species richness and Shannon-Wiener index (Shannon diversity), and total abundance (sum of maximum count of each species) were computed for each pair (sample site) by season.

Variations in bird diversity (species richness and Shannon diversity) and total abundance between zones were examined using a generalized linear model (GLM) and post-hoc analysis in R [40]. Distribution type used in GLMs (“glmmTMB” package) [41] differed between response variables or seasons due to overdispersion in the data. The GLMs of Shannon diversity were modeled with Gaussian distribution and the GLMs of total abundance with gamma distribution in all seasonal analyses. For species richness, Poisson distribution was used in winter and fall analyses and gamma distribution in spring analysis. We used Anova function in “car” package in order to determine the significance of overall effect of zones on each response variable in GLMs. Then, we compared least-squares means (lsmeans) and their 95% confidence intervals (CIs) between zones, which were estimated from GLMs, as post hoc analysis (“emmeans” package) [42]. We considered that lsmeans of zones were different at the *P* < 0.05 level if their 95% CIs did not overlap and the zonal effect was significant (Anova test, *P* < 0.05). Overdispersion was tested in “DHARMa” package [43].

To investigate how species’ traits were associated with differences in relative abundance across species between 5 zones, we adopted a fourth corner modeling that is implemented in traitglm function within “mvabund” package that employees a model-based approach for multivariate count data [44]. The fourth corner modeling requires environmental data (5 zones in our study), species’ abundance data, and species’ trait data. By using these data, it computes a matrix of trait-environment interaction coefficients, i.e., the fourth corner [44]. As species’ traits, we used body mass and foraging stratum which is related to water depth (ground [mudflat level], both ground and water surface, water surface, and below water surface). Trait information was compiled from Wilman et al. [45]. We tested whether species traits varied across zones in the fourth corner model using anova function which performs likelihood ratio test and bootstrapping (999 bootstraps for our analysis) in “mvabund” package. If the variation was significant, we generated a heat-map of the standardized fourth corner coefficients (FCs) from the model by applying LASSO penalty for easy interpretation. LASSO penalty removes interactions that do not explain variation in the model, i.e., insignificant interactions by setting them to zero [46]. The fourth corner analysis was also conducted by considering species’ family as a trait and pulling seasonal data together.

We performed Moran’s I test on the residuals of generalized linear models using “ape” package [47]. Spatial autocorrelation was negligible (*P* > 0.08). Residual plots did not show specific patterns.

#### Abundance pattern of conservation-related species (Csp)

Of 23 surveys, there was one survey that GIS records were inaccurate or missing. We excluded the survey and used data from the rest of surveys (22) for the analysis of Csp. Although we found 25 Csp, about half of them had few (≤10) observations across surveys and sites. Thus, we classified Csp into 2 species (Chinese egret, *Egretta eulophotes*, and Eurasian oystercatcher, *Haematopus ostralegus*) and 4 groups according to their family (spoonbill [Threskiornithidae], raptor [Accipitridae and Falconidae], sandpiper [sandpipers and their allies ‒ Scolopacidae]) and similarity in body size and observed distribution (crane/goose/stork). Four gull/tern species were not considered due to low number of observations even after grouping. One owl species was also excluded because it was detected only once and did not belong to any other group. All but three raptor species and Eurasian oystercatcher were migrants (S3 Table for species list and population status).

Geo-referenced abundance (count) data of each group and species were projected onto ArcGIS v. 10.3 (ESRI, Redlands, CA). From the coastline, we set a 2km buffer toward getbol and 1km buffer to inland as the size of area for the abundance distribution map. We determined the size to include all the GPS records but to avoid overprediction given that the most records were concentrated within ~ 1km of getbol side.

We emphasized that our primary interest was to visualize the abundance pattern of Csp along the coastline of Gochang getbol by making a spatially continuous abundance distribution map that can capture local variations in the pattern, rather than to predict abundance per se. We generated abundance distribution map for each group using one of spatial interpolation methods, i.e., the inverse distance weighted (IDW) interpolation implemented in geostatistical analysis tool in ArcGIS v. 10.3. Spatial interpolation methods estimate the value in unsampled sites in order to create spatially continuous data. IDW is commonly used because of its simplicity and ease of interpretation as prediction is strictly based on raw data, e.g., abundance value and distance between samples [48]. IDW assumes that “things that are close to one another are more alike than that are farther apart” [36]. The predictive value in unsampled point is more influenced by the value of closest sampled point than those farther away. That is, greater weights are given to points closest to prediction point and they decrease with increasing distance. The decreasing rate of weight depends on the power value (weighting parameter), *p*. Although prediction in IDW can be sensitive to local clusters and outliers, IDW assumes that this can be caused by local variations [49]. We log-transformed the abundance data for normalization after exploring normal Q-Q plots. We used different *p* (1, 2, and 3) and determined optimal *p* with two cross-validation statistics: Mean Error (ME) and Root Mean Square Error (RMSE). Prediction is considered unbiased when the ME is close to 0 and the RMSE is as small as possible. The ME and the RMSE at different *p* and optimal *p* used for IDW of each group are summarized in S4 Table.

## Results

Seventy-seven migratory bird species of 15 families were detected across 15 surveys and 10 sample sites (i.e., 20 points) used for community level analysis. Although species richness in winter (38) and fall (37) were similar, total abundance, i.e., sum of maximum count of each species was highest in winter: 13,002 and 4,628 individual birds in winter and fall, respectively. Spring showed lowest species richness (31) and abundance (2,226).

**Fig 2:**
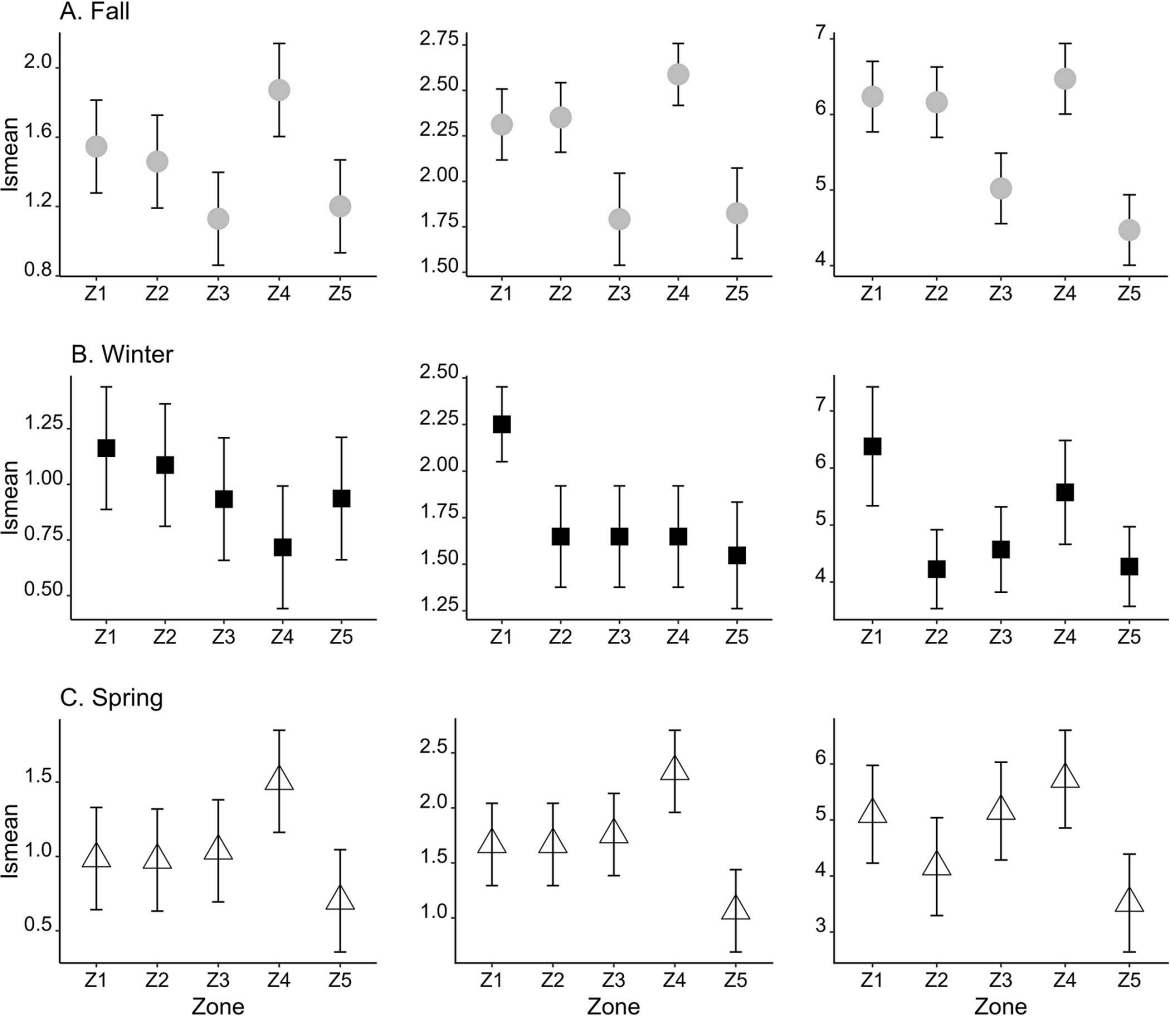
Least-squares means (lsmeans) and 95% confidence intervals of Shannon-Wiener diversity (left; Shannon diversity), species richness (middle), and total abundance (right) of migratory birds at each zone by season. Lsmeans (y-axis) and 95% confidence intervals (bars) are calculated from the generalized linear models.

Species diversity (species richness and Shannon diversity) and total abundance of migratory birds differed between zones (Anova test for GLM, *P* ≤ 0.02 in all seasonal analyses except Shannon diversity in winter; Table 2). Results of post-hoc analyses showed that differences in these indices between 5 zones varied by season (Fig 2). In fall, zone4 tended to have high Shannon diversity and species richness, especially significantly higher species diversity than zone3 and zone5 given their non-overlapping 95% CIs. Zone3 and zone5 showed lowest Shannon diversity and total abundance. However, in winter when bird composition was completely different from the other two seasons, Shannon diversity was consistently high at zone1. Total abundance was also greater at zone1compared to other zones except zone4. In spring, although significant differences in species diversity and total abundance were found between zone4 and zone5, total abundance tended to be lower at zone2 than zone4 considering slightly overlapping 95% CIs.

**Table 2:** Summary of Anova tests on the generalized linear models for the significance of differences in species diversity and total abundance between zones.

Season	Response variable	χ^2^	P
Fall	Shannon diversity	19.836	< 0.001
	Species richness	42.414	< 0.001
	Total abundance	47.091	< 0.001
Winter	Shannon diversity	6.2705	0.18
	Species richness	23.696	< 0.001
	Total abundance	20.45	< 0.001
Spring	Shannon diversity	11.614	0.02
	Species richness	23.349	< 0.001
	Total abundance	14.271	0.006

**Fig 3:**
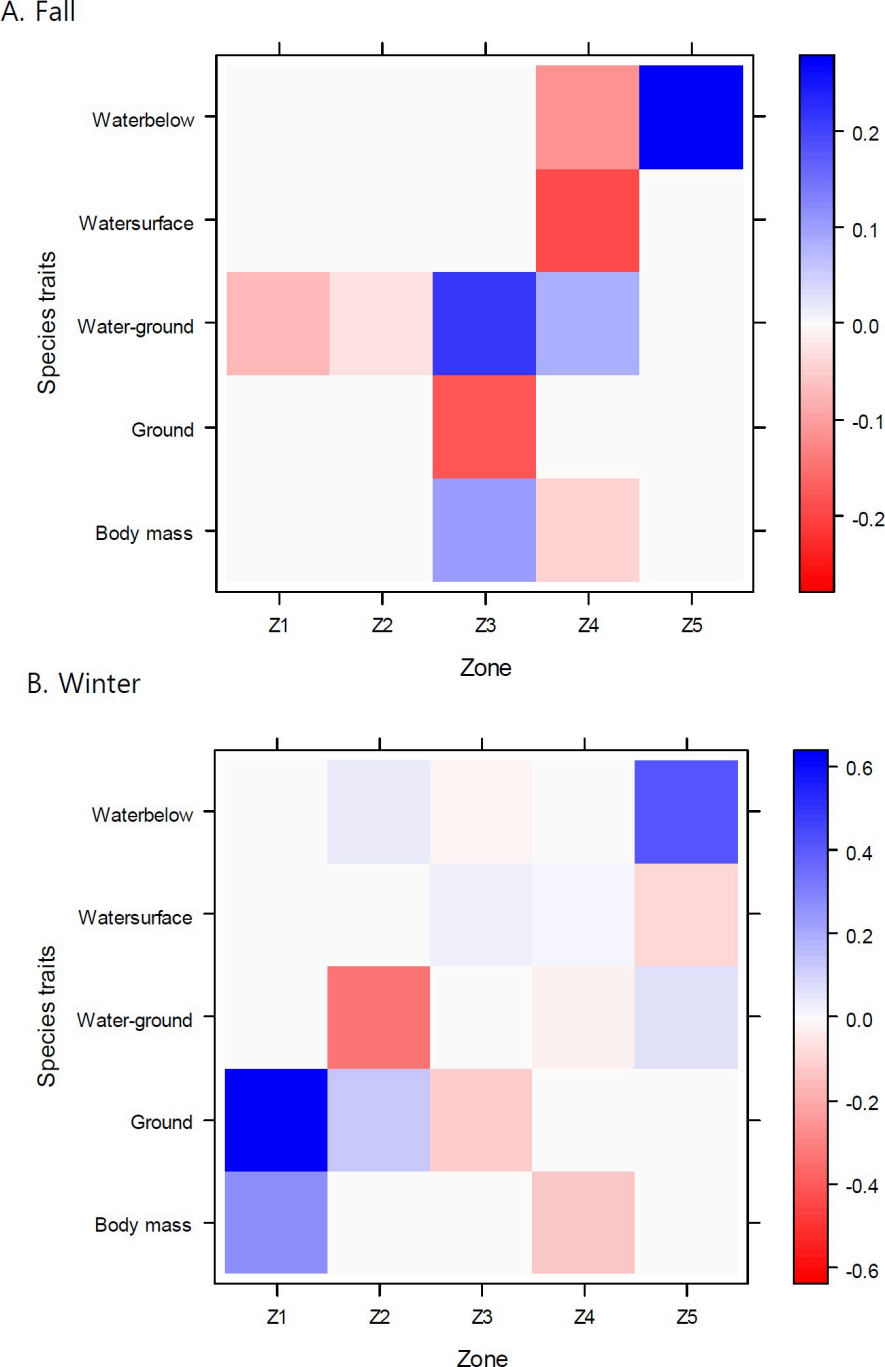
Association between species’ traits and 5 zones. Color bar on the right is the standardized fourth-corner coefficient (FC). Colors represent the strength (shading) and direction (blue = positive, white = no association, and red = negative) of the association.

Species’ traits also varied across zones in all seasons except spring (Likelihood ratio test, *P* = 0.001 in fall; *P* = 0.005 in winter; *P* = 0.389 in spring). The variation was of particular clear in winter (Fig 3). Zone1 showed positive associations with ground foraging species (strongly, FC = 0.64) and large species (FC = 0.27). This pattern was likely driven by cranes, geese/swans, and large raptors such as eagles and vultures abundant at zone1 (S2 and S3 Figs). Zone2 was negatively associated with species foraging at both water surface and ground levels (FC = -0.34), and weakly positively with ground foraging species (FC = 1.3). Zone5 showed positive association with species foraging below water in both winter (FC = 0.41) and fall (FC = 0.28), which were linked to high abundance of cormorants, grebes, and terns at zone5 (Fig 3, and S2 and S3 Figs). In fall, zone3 was negatively associated with ground foraging species (plovers; FC = -0.18) but positively with species foraging at both water surface and ground levels (some sandpipers; FC = 0.21) (Fig 3 and S2 Fig). Most associations between zone4 and foraging stratum and body mass traits were negligible in both seasons other than negative association with species foraging at both water surface and ground levels (FC = -0.19) in fall. However, zone4 had the strongest association with sandpipers (positive, FC = 0.35) across all families and zones (S2 Fig).

**Fig 4:**
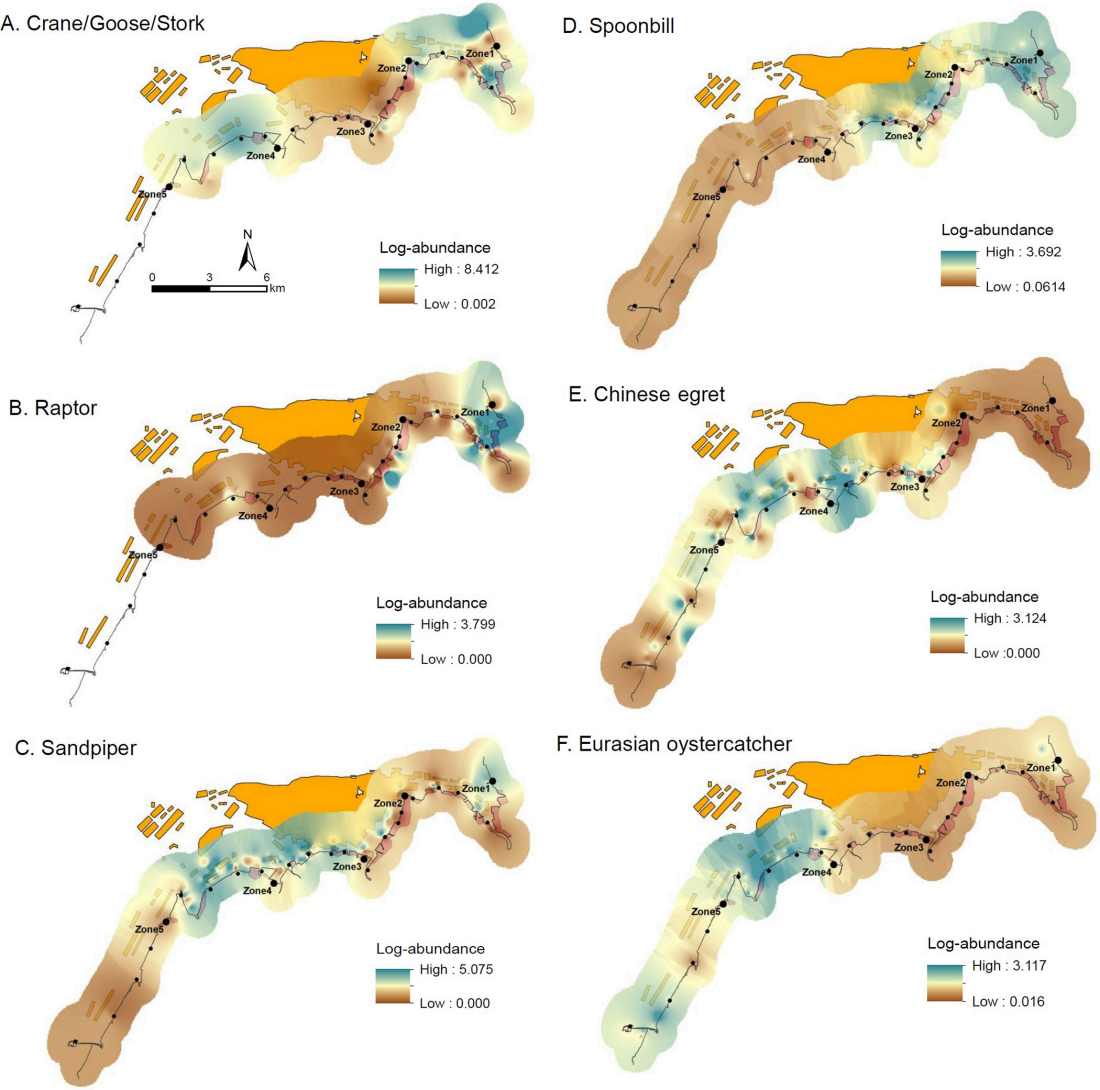
Abundance distribution map of conservation-related species generated by using the inverse distance weighted interpolation model. Of 6, 4 (A-D) groups are composed of 2 to 7 species (See methods and S3 Table). In the abundance maps of crane/goose/stork and raptor, zone5 is left in blank because of no detection. Orange and red colors on the background represent mudflat culture (mostly bivalve culture) and aquaculture pond (shrimp farming), respectively.

Abundance distribution map of Csp showed clear patterns (Fig 4): relatively high abundance of more Csp at zone4 and zone1 (4 Csp) than other three zones (1 or 2 Csp). Crane/goose/stork was abundant at zone1 followed by zone4. Raptors were largely concentrated at zone1, especially the 1^st^ and 2^nd^ point. Crane/goose/stork and raptor were not observed at zone5 and thus zone5 was excluded from the IDW interpolation model. Sandpipers were abundant at zone4, zone3 (mostly toward zone4), and the 1^st^ point of zone1. Spoonbills and Eurasia oystercatchers showed an opposite pattern. Spoonbills were abundant at zone1 and a few points of zone2 and zone3 but low at zone4 and zone5. Eurasian oystercatchers’ abundance was high at zone4 followed by zone5 (mostly between the 4^th^ and the 5^th^ point) but low at zone1, zone2, and zone3. High abundance of Chinese egret was also found at zone4 except an area between the 3^rd^ point and the 2^nd^ point where aquaculture pond was located.

## Discussion

Our study reveals spatial and temporal variations in species diversity and abundance of migratory birds in Gochang getbol and near areas: great species richness and/or Shannon diversity at zone1 (winter) and zone4 (fall and spring). It also shows the effect of species’ traits on relative abundance of species across zones: for example, positive association of ground [mudflat level] foraging species and large species with zone1 and sandpipers with zone4. Abundance distribution patterns of conservation-related species indicate high abundance of more species at zone1 and zone4 compared to other zones. Our findings suggest that less disturbed mudflats (i.e., little bivalve culture and aquaculture pond) adjacent to other natural/semi-natural habitats such as zone1 and 4 may be critical for the conservation of migratory birds in Gochang getbol where different levels of human activity occur across the getbol.

Habitat amount or habitat availability is one of the most important factors affecting diversity and population persistence of species [50]. Habitat loss, which is largely caused by numerous human activities, is considered a significant threat to global biodiversity [51, 52]. Human activities are also main causes of tidal flat habitat loss and degradation in the East Asian-Australasian Flyway and have driven a substantial decline of waterbird populations [6, 7, 9, 14, 16, 53]. Human activity such as clam harvesting and shellfish farming disturb bottom sediment and nutrient input [54]. Non-target macrobenthos can be accidently harvested in the process. Introduced commercial species of mollusks may increase a risk of homogenizing sediment communities [55]. These conditions affect migratory waterbird distribution by increasing mortality of benthic invertebrates and modifying benthic community composition [26, 27, 56, 57]. Human induced disturbances, e.g., vehicle riding and walking for clam harvest and recreational activities near coastline also interrupt foraging or roosting waterbirds, cause them to fly away, and increase energetic cost when they need to store fat and protein for their migrations [58, 59]. In our study, spatial distribution patterns of migratory birds are likely a result from the interplay of both factors, i.e., the amount of natural/semi-natural habitat and the intensity of human activity. Although zone3 has the largest exposed mudflat in the getbol, most of the mudflat is used for bivalve culture. Zone2 is similar to zone3 but bivalve culture occurs farther from the coastline than zone3, while its coastline is surrounded by aquaculture ponds where there used to be a mudflat. In other words, the amount of less disturbed mudflat is low at zone2 and zone3. Conversely, zone1 and zone4 show lower human activity than zone2 and zone3. Both zones also contain a larger amount of less disturbed mudflats as well as other natural/semi-natural habitats than zone 2, zone3, and zone5.

Natural/semi-natural habitat cover is of particular high and slightly heterogeneous at zone4 because of restored mudflat (tidal wetland or slough) and old salt pan. Salt pan is part of human activity like aquaculture pond; however, it can hold great species richness and abundance of shorebirds by serving as roosting sites and supplementary foraging sites especially during high tide [60]. The coastal area of zone4 also contains golf course that has small pond and vegetation, which may serve as artificial habitats [61]. Moreover, zone4 has heterogeneous sediment composition that changes dynamically with increasing distance from the coastline [36, 37]. Environmental heterogeneity including habitat and landscape heterogeneity often assumed to promote animal diversity [62, 63]. Habitat heterogeneity can drive bird species diversity in wetlands [64], have a positive effect on shorebird abundance when mudflat habitat is restricted [65], and increase functional diversity of macrobenthos [66]. In mudflat, abundance, biomass, and structure of the benthic macrofauna are influenced by sediment composition (e.g., silt or sand dominant) [67], which in turn affect the distribution of waterbirds [68–70]. Heterogeneous sediment composition in the mudflat area of zone4 can form a rich benthic community that may provide diverse food resources for a variety of migratory birds as found in Nanpu in the Yellow Sea region of China [71].

While the sediment of zone1 is mainly composed of silk and clay [36, 37], zone1 has environmental characteristics that could be favored by species preferring both mudflats and other natural/semi-natural habitats. At near the 1^st^ point, there are water channels running through croplands or connected to local lake as well as a tidal flat ecological park (wetland). The 2^nd^ and 3^rd^ points of zone1 are close to estuarine and vegetated water channel and lake, respectively. Inland area shows complex landscapes composed of small croplands and scattered woody patches. These features likely contribute to high abundance of raptors because of easy access to mudflat for hunting and available perching/nesting sites in inland areas, and less mudflat-dependent winter visitors such as cranes and geese/swans, leading to great diversity and abundance at zone1 in winter. However, the fine sediment type can limit the distribution of species favoring coarse sediment (i.e., sand), e.g., gulls [72]. Abundant raptors can also increase a predation risk to small sandpipers, influencing sandpipers’ habitat choice, behavior, distribution, and mortality [67, 73–75]. These features may be in part related to weakly negative association of zone1 with some gulls and sandpipers (S2 and S3 Figs).

Given the environmental complexity of Gochang getbol, the independent effect of bivalve culture and aquaculture on migratory birds is somewhat unclear. Our data (sample size and environmental data) also are too limited to examine the independent effect. However, abundance distribution of conservation-related species across the getbol may give us a clue. For example, Eurasian oystercatchers were concentrated at zone4 and zone5, with high abundance at zone4. These two zones have low or little bivalve culture, that is, bivalve density at zone4 and zone5 is low and particularly much lower compared to zone3 and zone2 considering the extent of bivalve culture concessions. Eurasian oystercatchers were also rare at zone1except near the 1^st^ point that does not have aquaculture pond. Godet et al. [26] described that intensive Manila clam culture, i.e., increasing Manila clam culture concessions degraded the quality of foraging ground for Eurasian oystercatchers and altered their spatial distribution. Schwemmer et al. [76] also found that the encounter rate of foraging Eurasian oystercatcher increased with bivalve density but decreased once the density exceeded a certain level. The distribution pattern of Eurasian oystercatchers in our study is in part consistent with the findings of these two studies, particularly Godet et al. [26], showing a decline in Eurasian oystercatchers with increasing bivalve culture.

It should also be noted that while conservation-related sandpipers (4 endangered or nearly threatened species that belong to Scolopacidae) were abundant at zone3, they were less abundant between the 1^st^ and the 3^rd^ points at which aquaculture ponds are located within zone3. Sandpipers were distributed at zone1 but only at the 1^st^ point with few or no aquaculture ponds, and rarely occurred at zone2 except one point. Even at zone4, their abundance was low at the area between the 2^nd^ and the 3^rd^ points where there is an aquaculture pond. Chinese egrets also showed low abundance at zone2 and little occurrence at zone1. Watetrbirds utilize artificial habitats including aquaculture ponds and salt pans/ponds in coastal areas [30, 60, 77–81]. They can also be abundant and diverse in areas with extensive aquaculture ponds [30]. However, most positive effect of these artificial habitats on waterbird diversity or population is often found in coastal areas where artificial habitats are dominant land cover. This is not the case of Gochang getbol which has relatively a small scale of aquaculture ponds compared to bivalve culture in getbol area and croplands on inland side. Although we cannot exclude the possibility that aquaculture ponds can be used as secondary foraging sites, our study indicates that some conservation-related species may avoid disturbed mudflats by aquaculture ponds or bivalve culture.

One may point out that high abundance of spoonbills (Black-faced spoonbill, *Platalea minor* and Eurasian spoonbill, *Platalea leucorodia*) at zone2 indicates a positive relationship between these two species and aquaculture ponds. Black-faced spoonbill favors habitats with shallow water and fine sediments without vegetation or other obstruction [82]. Eurasian spoonbill feed in estuaries on the wintering grounds [83] and use croplands and agricultural channels more often than Black-faced spoonbill [84]. Both zone1 and zone2 have habitat conditions favored by these species: fine sediment, and close location to estuary and rice field. Mudflat area of zone2 is more open compared to zone1, which explains higher occurrence and abundance of black-faced spoonbill at zone2 (25 detections with a maximum count of 71) than zone1 (17 detections with a maximum count of 55). We do not know whether spoonbills use aquaculture ponds, even after drained in winter due to lack of observations. However, the high abundance of spoonbills is more likely influenced by the sediment type and other environmental features rather than aquaculture ponds.

Of 5 zones, zone5 has the smallest amount of mudflat and its sediment is predominated by sand, i.e., homogenous. This could restrict prey type and abundance and hence waterbird species occurring at zone5. While the level of bivalve culture and aquaculture is low, zone5 has a beach area that attracts tourists. It is not surprising that species diversity and abundance of most migratory birds tended to be low at zone5 regardless of seasons. However, critically endangered species, Chinese crested tern was observed in zone5 only and twice during our conservation-related species survey. Although zone5 cannot be an important habitat to promote migratory bird diversity, we should not ignore its habitat value for Chinese crested tern in habitat management plan of Gochang getbol.

## Conclusion

Overall, our findings can be useful to determine priority areas for conservation management and habitat restoration in Gochang getbol. Zone4 area can be valuable to conserve species diversity of migratory waterbirds during spring and fall seasons and populations of conservation-related species including sandpipers, whereas zone1 area can be a critical habitat for wintering migratory birds including large waterbirds and raptors. They also imply the importance of preserving mudflats that are less disturbed by human activity and adjacent to other natural/semi-natural habitats.

However, there are several aspects that we need further investigations. First, our survey was centered on mudflat side, and performed surveys between mid-tide and high tide, starting ~ 2 hours and ending before high tide. We do not have observations about where birds rest, forage, and roost in other areas outside the mudflat. Tide time affects foraging site preference of waterbirds [85]. Waterbirds forge largely at mudflat in low tide when the mudflat is exposed most but can utilize other natural or artificial habitats (e.g., wetlands, salt pans, aquaculture ponds) in high tide. This pattern can also vary depending on species and/or season, making it difficult to determine suitable tide time for survey [86, 87]. Although our survey time is commonly used in monitoring programs of waterbirds, we acknowledge that the patterns found in our study, particularly species abundance might be biased by survey time and mudflat-centered survey. Second, the size of exposed mudflats could affect bird detection. Exposed mudflat of zone3 is the largest in the getbol. Birds foraging far from the coastline are unlikely detected in our survey. This may result in relatively low detections of individuals and species at zone3. Further study is needed to test the imperfect detection by adopting secondary methods such as unmanned aerial vehicles [88, 89] or including additional survey sites, i.e., Chenier and small island in the getbol. Lastly, there has been a conflict between conservation and local economy in Gochang getbol because local people’ livelihoods largely depend on their resource use in the getbol. Our findings show a possible link between resource use by human and conservation-related species although it is indirect. However, our study focused on conservation issue. It does not convey information on the other issue, i.e., foraging or utilization rate of migratory birds on bivalves and shrimps cultured for harvest and its potential economic impact on local people. There is a strong need for future study to explore this issue, which is essential to develop a management plan that can mitigate the conflict as well as ensure the sustainability of the Gochang getbol.

## Supporting information

S1 Table Percent cover of landscape elements within a 2km–circular buffer surrounding a sample point. (DOCX)

S2 Table List of species and their traits. (DOCX)

S3 Table Conservation-related species considered for generating abundance distribution map. (DOCX)

S4 Table Cross-validation results of IDW (inverse distance weighted) interpolation for each group of conservation related species (Csp) with 3 levels of *p* (Power). (DOCX)

S1 Fig Land cover of Gochang getbol and surroudning areas in 1999, 2009, and 2019. (DOCX)

S2 Fig The result of fourth corner analysis by considering species’ family as a trait and pulling together the bird data of three seasons. (DOCX)

S3 Fig Relationships between species abundance and 5 zones. (DOCX)

S1 File Bird data used for community level analysis. (XLSX)
