# Supplementary material for: Spatiotemporal variations in migratory bird diversity and abundance along the coast of Gochang getbol
Source: PLoS One. 2024 May 31;19(5):e0300353. doi: 10.1371/journal.pone.0300353 (PMC11142517; doi:10.1371/journal.pone.0300353)
Supplement: S1 Table — (DOCX) [file pone.0300353.s001.docx]

S1 Table. Percent cover of landscape elements within a 2km –circular buffer surrounding a sample point. The 5^th^ point of each zone is highlighted in red. Note the difference in the percent cover of mudflat culture or mudflat between the 5^th^ point and the other points within a zone, particularly at zone1, 2, and 3.

| Zone | Point | Aquaculture | Mudflat culture | Mudflat | Wetland | Salt pan | Agriculture |
| --- | --- | --- | --- | --- | --- | --- | --- |
| Zone1 | 1 | 5.56 | 2.9 | 37.64 | 3.85 | 0 | 36.57 |
|  | 2 | 9.42 | 1.64 | 18.14 | 0.23 | 0 | 60.15 |
|  | 3 | 4.99 | 5.38 | 50.64 | 0.21 | 0 | 27.71 |
|  | 4 | 5.49 | 9.02 | 46.97 | 0.1 | 0 | 27.24 |
|  | 5 | 8.46 | 24.62 | 29.26 | 0.03 | 0 | 27.59 |
| Zone2 | 1 | 8.09 | 32.82 | 28.79 | 0 | 0 | 20.57 |
|  | 2 | 10.4 | 30.01 | 23.02 | 0 | 0 | 31.23 |
|  | 3 | 11.86 | 22.36 | 23.24 | 0 | 0 | 34.15 |
|  | 4 | 12.27 | 23.19 | 23.81 | 0 | 0 | 31.29 |
|  | 5 | 7.51 | 4.71 | 12.99 | 0.33 | 0 | 24.86 |
| Zone3 | 1 | 8.35 | 16.48 | 17.16 | 0.14 | 0 | 24.43 |
|  | 2 | 6 | 34.54 | 16.11 | 0.01 | 0 | 18.55 |
|  | 3 | 4.03 | 37.1 | 14.49 | 0 | 0 | 20.93 |
|  | 4 | 2.6 | 36.1 | 25.41 | 1.32 | 0 | 22.31 |
|  | 5 | 2.71 | 16.33 | 36.96 | 3.38 | 0 | 30.75 |
| Zone4 | 1 | 2.95 | 1.83 | 26.91 | 3.38 | 0 | 46.11 |
|  | 2 | 2.95 | 4.28 | 49.48 | 3.38 | 1.78 | 29.57 |
|  | 3 | 2.36 | 5.54 | 51.95 | 2.14 | 8.36 | 21.16 |
|  | 4 | 1.79 | 5.6 | 42.74 | 0 | 15.91 | 12.57 |
|  | 5 | 2.51 | 4.95 | 28.51 | 0 | 1.35 | 14.02 |
| Zone5 | 1 | 1.15 | 6.18 | 12.91 | 0 | 0 | 32.39 |
|  | 2 | 1.14 | 5.61 | 5.51 | 0.07 | 0 | 37.64 |
|  | 3 | 0.48 | 3.01 | 4.99 | 0.07 | 0 | 42.89 |
|  | 4 | 0 | 3.69 | 4.37 | 0 | 0 | 37.6 |
|  | 5 | 0 | 1.44 | 3.99 | 0 | 0 | 7.61 |
