# Supplementary material for: Spatiotemporal variations in migratory bird diversity and abundance along the coast of Gochang getbol
Source: PLoS One. 2024 May 31;19(5):e0300353. doi: 10.1371/journal.pone.0300353 (PMC11142517; doi:10.1371/journal.pone.0300353)
Supplement: S2 Table — (DOCX) [file pone.0300353.s002.docx]

S2 Table. List of species and their traits. Abundance is represented by maximum count. Abbreviation: Waterbelow, foraging under water (e.g., diving birds); Watersur, foraging at shallow water or water surface; Watergr, foraging at both ground level and shallow water; Ground, foraging at ground level (mudflat); aboveGr, foraging at higher place than ground, e.g., shrub and tree; sv, summer visitor; pm, passenger; wv, winter visitor.

| Season | Scientific | English | Body Mass | Foraging stratum | Migratory status | Abundance  (Fall/Spring) |
| --- | --- | --- | --- | --- | --- | --- |
| Fall/Spring | Actitis hypoleucos | Common Sandpiper | 48 | Watgr | sv | 4/1 |
| Fall/Spring | Alcedo atthis | Common Kingfisher | 31.09 | Waterbelow | sv | 1/2 |
| Fall/Spring | Ardea alba | Great Egret | 871.33 | Watgr | sv | 215/13 |
| Fall/Spring | Ardea cinerea | Grey Heron | 1443 | Watersur | sv | 159/15 |
| Fall/Spring | Arenaria interpres | Ruddy Turnstone | 135.98 | Ground | pm | 26/33 |
| Fall | Butorides striata | Striated Heron | 201.5 | Watersur | sv | 1 |
| Fall/Spring | Calidris alba | Sanderling | 51.71 | Ground | pm | 70/1 |
| Fall/Spring | Calidris canutus | Red Knot | 141.87 | Ground | pm | 8/45 |
| Fall/Spring | Calidris ruficollis | Red-necked Stint | 26.79 | Watgr | pm | 82/152 |
| Fall/Spring | Calidris tenuirostris | Great Knot | 192 | Ground | pm | 161/251 |
| Fall/Spring | Charadrius alexandrinus | Kentish Plover | 42.3 | Ground | pm | 504/73 |
| Spring | Charadrius dubius | Little Ringed Plover | 38.7 | Ground | sv | 2 |
| Fall/Spring | Charadrius mongolus | Lesser Sand Plover | 64 | Ground | pm | 1219/430 |
| Fall/Spring | Egretta eulophotes | Chinese Egret | 461.83 | Watersur | sv | 34/8 |
| Fall/Spring | Egretta garzetta | Little Egret | 312 | Watersur | sv | 21/1 |
| Fall | Egretta intermedia | Intermediate Egret | 458.83 | Watersur | sv | 10 |
| Fall | *Falco subbuteo | Eurasian Hobby | 208.17 | aboveGr | sv | 1 |
| Fall/Spring | Heteroscelus brevipes | Grey-tailed Tattler | 127 | Watgr | pm | 65/31 |
| Fall | Larus saundersi | Saunders's Gull | 198 | Watgr | sv | 17 |
| Fall/Spring | Limicola falcinellus | Broad-billed Sandpiper | 37.1 | Ground | pm | 2/1 |
| Fall/Spring | Limosa lapponica | Bar-tailed Godwit | 291.65 | Watgr | pm | 160/128 |
| Fall/Spring | Limosa limosa | Black-tailed Godwit | 288.37 | Watgr | pm | 260/17 |
| Fall/Spring | Numenius arquata | Eurasian Curlew | 802.99 | Ground | pm | 37/7 |
| Fall/Spring | Numenius madagascariensis | Far Eastern Curlew | 792 | Ground | pm | 194/56 |
| Fall/Spring | Numenius phaeopus | Whimbrel | 364.57 | Ground | pm | 187/223 |
| Fall/Spring | Pandion haliaetus | Osprey | 1483.2 | Watersur | pm | 2/1 |
| Fall/Spring | Platalea minor | Black-faced Spoonbill | 1228 | Watersur | sv | 78/28 |
| Fall/Spring | Pluvialis fulva | Pacific Golden Plover | 134.9 | Ground | pm | 9/5 |
| Fall/Spring | Pluvialis squatarola | Grey Plover | 250 | Ground | pm | 152/200 |
| Spring | Sterna albifrons | Little Tern | 57 | Waterbelow | sv | 13 |
| Fall | Sterna hirundo | Common Tern | 129.15 | Waterbelow | pm | 3 |
| Fall | Tringa erythropus | Spotted Redshank | 158 | Watersur | pm | 12 |
| Fall/Spring | Tringa glareola | Wood Sandpiper | 62.05 | Watgr | pm | 2/2 |
| Fall | Tringa guttifer | Spotted Greenshank | 158 | Watgr | pm | 2 |
| Fall/Spring | Tringa nebularia | Common Greenshank | 187 | Watersur | pm | 405/35 |
| Fall/Spring | Tringa ochropus | Green Sandpiper | 71.4 | Watgr | pm | 1/2 |
| Fall/Spring | Tringa stagnatilis | Marsh Sandpiper | 77.5 | Watersur | pm | 43/3 |
| Fall | Tringa totanus | Common Redshank | 129 | Watersur | pm | 30 |
| Fall/Spring | Xenus cinereus | Terek Sandpiper | 78.8 | Watgr | pm | 451/447 |
| Winter | Aegypius monachus | Cinereous Vulture | 9320.55 | Ground | wv | 29 |
| Winter | Anas acuta | Northern Pintail | 944.62 | Watersur | wv | 590 |
| Winter | Anas clypeata | Northern Shoveler | 612.56 | Watersur | wv | 4 |
| Winter | Anas crecca | Common Teal | 341.89 | Watersur | wv | 80 |
| Winter | Anas falcata | Falcated Duck | 645.83 | Watersur | wv | 7 |
| Winter | Anas formosa | Baikal Teal | 433.98 | Watersur | wv | 50 |
| Winter | Anas penelope | Eurasian Wigeon | 770.03 | Watgr | wv | 60 |
| Winter | Anas platyrhynchos | Mallard | 843.42 | Watersur | wv | 952 |
| Winter | Anas strepera | Gadwall | 915.58 | Watersur | wv | 19 |
| Winter | Anser albifrons | Greater White-fronted Goose | 2506.39 | Ground | wv | 3700 |
| Winter | Anser fabalis | Bean Goose | 2754.73 | Ground | wv | 5000 |
| Winter | Asio flammeus | Short-eared Owl | 322.61 | Ground | wv | 1 |
| Winter | Aythya ferina | Common Pochard | 822.99 | Watersur | wv | 120 |
| Winter | Aythya fuligula | Tufted Duck | 701.17 | Waterbelow | wv | 8 |
| Winter | Aythya marila | Greater Scaup | 1005.37 | Waterbelow | wv | 2 |
| Winter | Bucephala clangula | Common Goldeneye | 918.56 | Waterbelow | wv | 10 |
| Winter | Buteo buteo | Common Buzzard | 759.1 | Ground | wv | 2 |
| Winter | Calidris alpina | Dunlin | 51.89 | Ground | wv | 1880 |
| Winter | Ciconia boyciana | Oriental Stork | 4847.74 | Watersur | wv | 7 |
| Winter | Cygnus cygnus | Whooper Swan | 9349.99 | Watersur | wv | 4 |
| Winter | Fulica atra | Common Coot | 717.12 | Watersur | wv | 5 |
| Winter | Grus monacha | Hooded Crane | 3729.9 | Watgr | wv | 54 |
| Winter | Haliaeetus albicilla | White-tailed Eagle | 4729.27 | Watgr | wv | 2 |
| Winter | Larus argentatus | Herring Gull | 1090.99 | Watgr | wv | 600 |
| Winter | Larus cachinnans | Caspian Gull | 1111.89 | Watgr | wv | 11 |
| Winter | Larus heuglini | Lesser Black-backed Gull | 762.36 | Watgr | wv | 10 |
| Winter | Larus hyperboreus | Glaucous Gull | 1529.04 | Watgr | wv | 1 |
| Winter | Larus ridibundus | Black-headed Gull | 284 | Watgr | wv | 26 |
| Winter | Larus schistisagus | Slaty-backed Gull | 1323 | Watgr | wv | 1 |
| Winter | Mergellus albellus | Smew | 608.55 | Waterbelow | wv | 2 |
| Winter | Mergus merganser | Common Merganser | 1451.02 | Waterbelow | wv | 44 |
| Winter | Mergus serrator | Red-breasted Merganser | 1015.17 | Waterbelow | wv | 15 |
| Winter | Phalacrocorax capillatus | Japanese Cormorant | 2801.78 | Waterbelow | wv | 3 |
| Winter | Phalacrocorax carbo | Great Cormorant | 2528.97 | Waterbelow | wv | 75 |
| Winter | Platalea leucorodia | Eurasian Spoonbill | 1868 | Watersur | wv | 13 |
| Winter | Podiceps cristatus | Great Crested Grebe | 730.96 | Waterbelow | wv | 10 |
| Winter | Tadorna tadorna | Common Shelduck | 1146.83 | Watersur | wv | 42 |
| Winter | Vanellus vanellus | Northern Lapwing | 218.37 | Ground | wv | 5 |

*Species not included in the fourth-corner analysis because they are the only ones that can be classified into above ground foraging stratum with very few detections.
