# Supplementary material for: Spatiotemporal variations in migratory bird diversity and abundance along the coast of Gochang getbol
Source: PLoS One. 2024 May 31;19(5):e0300353. doi: 10.1371/journal.pone.0300353 (PMC11142517; doi:10.1371/journal.pone.0300353)
Supplement: S3 Table — (DOCX) [file pone.0300353.s003.docx]

S3 Table. Conservation-related species considered for generating abundance distribution map. Sandpiper includes sandpipers and their allies that belong to Scolopacidae. Raptor has two family (Accipitridae and Falconidae).

| Group | Scientific name | Common name | Population status | # of detections |
| --- | --- | --- | --- | --- |
| Sandpiper | *Calidris tenuirostris* | Great Knot | Endangered | 29 |
|  | *Numenius madagascarien* | Far Eastern Curlew | Endangered | 78 |
|  | *Tringa guttifer* | Spotted Greenshank | Endangered | 1 |
|  | *Limosa lapponica* | Bar-tailed Godwit | Nearly threatened | 40 |
| Crane/Goose/Stork | *Cygnus cygnus* | Wooper Swan | Least concern | 1 |
|  | *Anser fabalis* | Bean Goose | Least concern | 19 |
|  | *Ciconia boyciana* | Oriental Stork | Endangered | 10 |
|  | *Grus monacha* | Hooded Crane | Vulnerable | 9 |
| Chinese egret | *Egretta eulophotes* | Chinese Egret | Vulnerable | 105 |
| Spoonbill | *Platalea leucorodia* | Eurasian Spoonbill | Least concern | 29 |
|  | *Platalean minor* | Black-faced Spoonbill | Endangered | 87 |
| Eurasian oystercatcher | *Haematopus ostralegus^1^* | Eurasian Oystercatcher | Nearly threatened | 82 |
| Raptor | *Aegypius monachus* | Cinereous Vulture | Nearly threatened | 18 |
|  | *Falco peregrinus^1^* | Peregrine Falcon | Least concern | 15 |
|  | *Pandion haliaetus* | Osprey | Least concern | 8 |
|  | *Accipiter nisus^1^* | Eurasian Sparrowhawk | Least concern | 2 |
|  | *Falco subbuteo* | Eurasian Hobby | Least concern | 2 |
|  | *Circus cyaneus^2^* | Hen Harrier | Least concern | 3 |
|  | *Falco tinnunculus^1^* | Common Kestrel | Least concern | 19 |
|  | *Haliaeetus albicilla* | White-tailed Eagle | Least concern | 10 |

^1^Resident species

^2^Species detected during the visits but excluded for community level analyses
