# Supplementary material for: Spatiotemporal variations in migratory bird diversity and abundance along the coast of Gochang getbol
Source: PLoS One. 2024 May 31;19(5):e0300353. doi: 10.1371/journal.pone.0300353 (PMC11142517; doi:10.1371/journal.pone.0300353)
Supplement: S4 Table — (DOCX) [file pone.0300353.s004.docx]

S4 Table. Cross-validation results of IDW (inverse distance weighted) interpolation for each group of conservation related species (Csp) with 3 levels of *p* (Power). When ME lose to 0 and RMSE is as low as possible, prediction is considered unbiased. Optimal *p* selected for final IDW interpolation is bolded. Abbreviation: ME, mean error; RMSE, root mean squared error.

| Csp group | Index | Power | | |
| --- | --- | --- | --- | --- |
|  |  | 1 | 2 | 3 |
| Crane/goose/stork | ME | -0.1766 | **-0.1063** | -0.8725 |
|  | RMSE | 2.1863 | **2.0749** | 2.1806 |
| Spoonbill | ME | **0.1670** | 0.1933 | 0.1874 |
|  | RMSE | **1.2797** | 1.3171 | 1.3435 |
| Chinese egret | ME | 0.0391 | **0.0306** | 0.0332 |
|  | RMSE | 0.8549 | **0.9147** | 0.9697 |
| Eurasian oystercatcher | ME | **0.0313** | 0.0468 | 0.0694 |
|  | RMSE | **0.9850** | 1.0554 | 1.0991 |
| Raptor | ME | 0.0457 | 0.0203 | **0.0068** |
|  | RMSE | 0.8544 | 0.8402 | **0.8444** |
| Sandpiper | ME | 0.1364 | 0.1070 | **0.0928** |
|  | RMSE | 1.4855 | 1.5472 | **1.6012** |
