# Supplementary material for: Spatiotemporal variations in migratory bird diversity and abundance along the coast of Gochang getbol
Source: PLoS One. 2024 May 31;19(5):e0300353. doi: 10.1371/journal.pone.0300353 (PMC11142517; doi:10.1371/journal.pone.0300353)

S1 Figure. Land cover of Gochang getbol and surroudning areas in 1999, 2009, and 2019. In the national land cover, salt ponds are considerd as mudflat/wetland and “Others” inlcudes restored tidal slough, sand, and so on. Satellite images assocaited with the maps was not taken in low tide. Thus, the actaul amount of mudflat is much higher than that shown in this figure, wherease the amount of west sea/water is much lower (see Fig 1.). Note the signficant increase in aquaculture (shrimp farming) along the coastline of the getbol between 1999 and 2009.


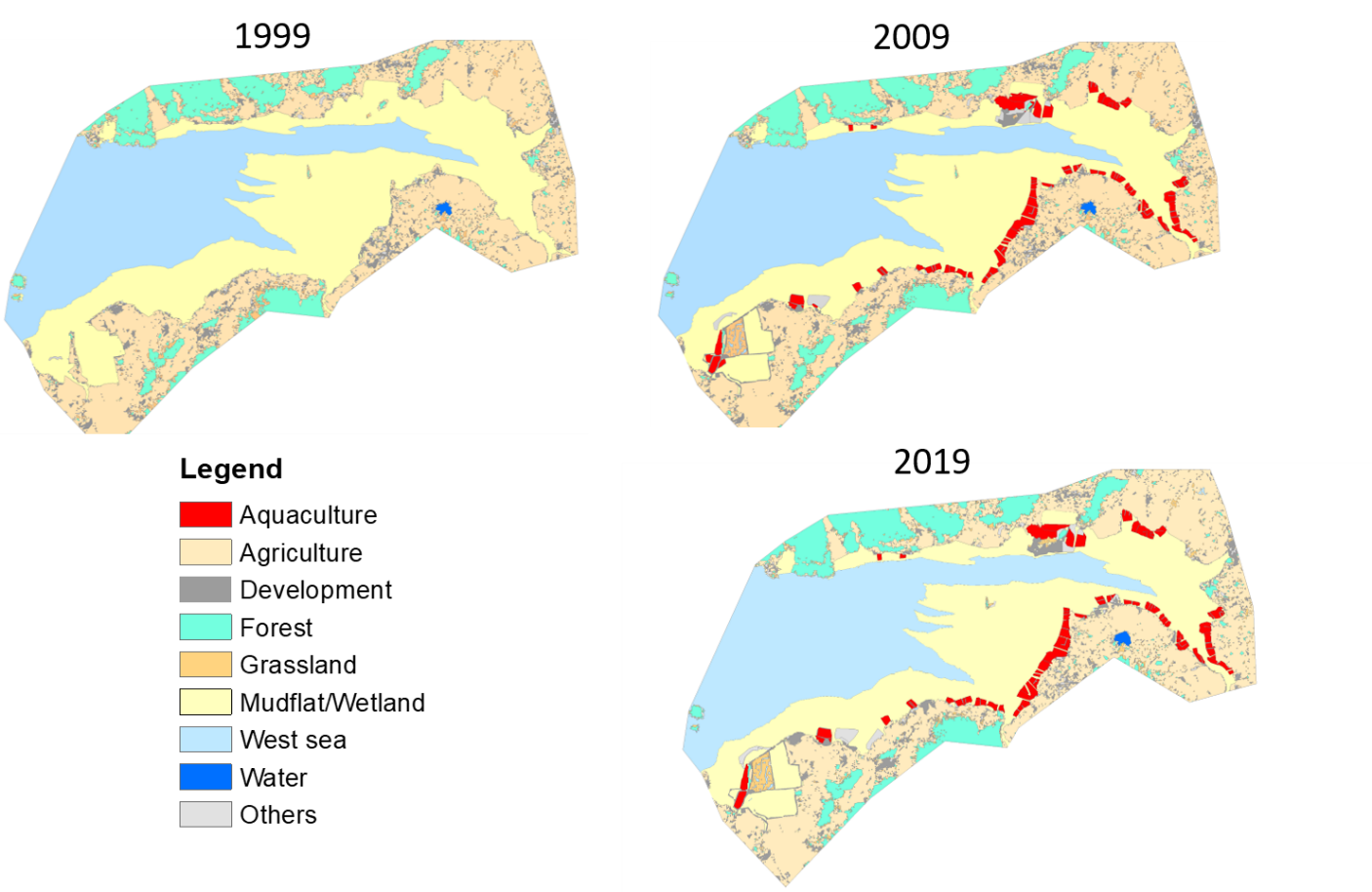

Supplement: S1 Fig — (DOCX) [file pone.0300353.s005.docx]
