# Supplementary material for: Spatiotemporal variations in migratory bird diversity and abundance along the coast of Gochang getbol
Source: PLoS One. 2024 May 31;19(5):e0300353. doi: 10.1371/journal.pone.0300353 (PMC11142517; doi:10.1371/journal.pone.0300353)
Supplement: S2 Fig — (DOCX) [file pone.0300353.s006.docx]

S2 Figure. The result of forth-corner analysis by considering species’ family as a trait and pulling together the bird data of three seasons. Raptor1 and 2 include osprey/kite/hawk/eagle (Accipitridae) and falcon (Falconidae), respectively. Sandpiper is composed of sandpipers and their allies belonging to Scolopacidae. Y-axis represent the fourth-corner coefficient that shows the strength(shading) and direction (blue = positive, white = no association, and red = negative) of relationship between species’ family and zones.
