# Supplementary material for: Spatiotemporal variations in migratory bird diversity and abundance along the coast of Gochang getbol
Source: PLoS One. 2024 May 31;19(5):e0300353. doi: 10.1371/journal.pone.0300353 (PMC11142517; doi:10.1371/journal.pone.0300353)
Supplement: S3 Fig — (DOCX) [file pone.0300353.s007.docx]

S3 Figure. Relationships between species abundance and 5 zones. Colors on the right represent the strength (shading) and direction (blue = positive, white = no association, and red = negative) of their relationships. Asterisk (*) indicates conservation-related species.

B. Winter

A. Fall

*

*
